# Supplementary material for: The transcriptional landscape of the cultured murine middle ear epithelium in vitro
Source: Biol Open. 2021 Apr 23;10(4):bio056564. doi: 10.1242/bio.056564 (PMC8084567; doi:10.1242/bio.056564)
Supplement: Supplementary information [file biolopen-10-056564-s1.pdf]

Table S1. Differential gene expression of MEECs Day 0 vs original

Click here to download Table S1

Table S2. Differential gene expression of MEECs Day 7 vs Day 0

Click here to download Table S2

Table S3. Differential gene expression of MEECs Day 7 vs original

Click here to download Table S3

Biology Open • Supplementary information
